# Midline supracerebellar infratentorial approach with clip occlusion of a straight sinus dural arteriovenous fistula

**DOI:** 10.3171/2025.7.FOCVID2586

**Published:** 2025-10-01

**Authors:** Anna L. Huguenard, Visish M. Srinivasan, Mohamed A. Labib, Jakub Godzik, Michael T. Lawton

**Affiliations:** Department of Neurosurgery, Barrow Neurological Institute, St. Joseph’s Hospital and Medical Center, Phoenix, Arizona

**Keywords:** dural arteriovenous fistula, straight sinus, supracerebellar infratentorial approach

## Abstract

Straight sinus dural arteriovenous fistulas (dAVFs), classified as tentorial type 2, can be approached with a torcular craniotomy and supracerebellar infratentorial approach, with gravity retraction from a sitting position optimizing the view. This video presents the case of a man in his early 60s with a thunderclap headache. Imaging showed a subarachnoid hemorrhage, and angiography confirmed a straight sinus dAVF. Endovascular embolization reduced flow, but further obliteration with surgical ligation was required. Intraoperative identification of the arterialized veins led to the fistula, and 2 efferent veins were clipped and divided. The patient tolerated the procedure well without new deficits, and angiography confirmed dAVF occlusion.

The video can be found here: https://stream.cadmore.media/r10.3171/2025.7.FOCVID2586

## Transcript

This video will demonstrate the midline supracerebellar infratentorial approach with clip occlusion of a straight sinus dural arteriovenous fistula.

### 0:30 Clinical Presentation.

The patient is in his early 60s. He presented with a thunderclap headache. His neurological examination was unremarkable. A CT scan demonstrated subarachnoid hemorrhage, and an angiogram confirmed a straight sinus dural arteriovenous fistula. In the classification of tentorial dural arteriovenous fistulas, this is a type 2.^1^

### 0:54 Preoperative Imaging.

Here are his CT angiogram images in coronal and sagittal views, showing the dilated veins that are centered around the torcular region but on further evaluation appear to originate right from the straight sinus just proximal to the torcular region. You can see the veins draining the fistula have these variceal dilatations. Here are his angiographic images, which show feeding of the fistula from the extracranial dural supply as well as from the tentorial artery. Note the dilated venous varices that drain the fistula. The patient underwent embolization, but the embolization was unsuccessful in obliterating the fistula.^2^

### 1:42 Patient Positioning.

Therefore, surgery was needed, and the surgical approach was a supracerebellar infratentorial approach in the sitting position to allow gravity to help open the supracerebellar plane. These are illustrations showing the patient in sitting position, and on the right, you can see the torcular craniotomy, which extends above the torcular region to fully expose the torcula.

### 2:09 Surgical Strategy.

Here are the key surgical strategic steps. First was preoperative embolization^3^ with Onyx to reduce flow through the fistula. Next was the torcular craniotomy in the sitting position. Gravity retraction was needed to open up the supracerebellar plane. The identification of two arterialized veins coming from the straight sinus was critical. The fistula site was confirmed with IC green videoangiography. The two efferent veins were clip occluded, cauterized, and divided. IC green videoangiography was again used to confirm no further shunting.

### 2:49 Intraoperative Video.

Here is the intraoperative video, and you can see, with the patient in the sitting position, we have gravity retracting the cerebellum. You can see Gelfoam over the torcular region for hemostasis, and this provides us with perfect access to the supracerebellar plane. The patient presented with subarachnoid hemorrhage, so you see blood in that supracerebellar space. But as we dissect deeper, we come upon the arterialized venous trunk. You can see that telltale discoloration of the vein with whitening of the wall and this classic vasa vasorum seen within the wall of the vein. Working around the venous structures, we can see the fistula’s site. This is just some additional dissection to free up the left-sided cerebellar hemisphere and widen our exposure. Here is a normal vein coming into view to the patient’s left of the fistula. Now you have a nice overview of the dural AV fistula, and in this particular case, there are two outflow veins. This is atypical, as usually the fistulas drain to a single vein. This IC green study shows you the circuitous pathway of this arterialized outflow. Here now the veins are ready for clip occlusion. I’ll first clip this more posteriorly located vein. I’m using a long aneurysm clip to occlude the vein just as it exits the straight sinus. And with the clip on, I can cauterize here and divide the vein to completely interrupt this connection. You see some Onyx within the lumen there. This takes care of the first of the two veins. We now turn our attention more anteriorly to this deeper vein, and again the vein is clipped, then cauterized, then incised to disconnect it. You can see that we have an easy time of doing this maneuver and seeing everything, because gravity is opening up this space for us. Here is the normal vein, which we’ll preserve in order to allow a normal venous drainage of the cerebellum. The IC green videoangiogram is repeated here in order to confirm that the shunt flow is no longer there. Here is an overview, again showing the clips in place, the dural flap elevating the torcula, and the exposure gained with this craniotomy in the sitting position. Also, you can note how the veins that were once arterialized are darkened in color.

### 5:27 Outcome.

The patient tolerated this procedure well. He was monitored in the ICU for vasospasm postoperatively. His postoperative angiogram demonstrated complete obliteration of the dural arteriovenous fistula.

### 5:41 Postoperative Imaging.

Here are his postoperative images. On the left, you can see a scout x-ray, which shows that the torcular craniotomy was elevated in two pieces.^4^ By doing the suboccipital craniotomy first, I was able to strip the torcular dura from the inner table of the second piece, which was then raised as a separate second craniotomy. You can see on the postoperative scout films how these two pieces plate together very easily. The angiogram on the right demonstrates that the dural AV fistula no longer shunts.

### 6:17 Conclusions.

In conclusion, straight sinus dural arteriovenous fistulas are one of the more common tentorial dural AV fistulas. This is a type 2 fistula, which fits into this classification with galenic, straight sinus, torcular, tentorial, superior petrosal sinus, and incisural fistulas. The supracerebellar infratentorial approach in the sitting position allows gravity to open this supracerebellar plane and facilitate the exposure. Torcular craniotomy in elderly men in the sitting position may require this two-piece craniotomy to protect the dural sinuses. Most dural AV fistulas have a single efferent vein, whereas this one had two. Venous interruption makes microsurgical treatment simple and safe.^5^ Thank you.

## Acknowledgments

We thank the staff of Neuroscience Publications at Barrow Neurological Institute for assistance with manuscript and video preparation.

## Disclosures

Dr. Huguenard reported stock ownership in and board member of Aurenar outside the submitted work. Dr. Godzik reported personal fees from ATEC, DePuy Synthes, Kuros, and SignaL outside the submitted work.

## Author Contributions

Primary surgeon: Lawton. Assistant surgeon: Huguenard, Srinivasan, Labib. Editing and drafting the video and abstract: Lawton, Huguenard, Srinivasan. Critically revising the work: Lawton, Huguenard, Srinivasan. Reviewed submitted version of the work: Lawton, Huguenard, Srinivasan, Godzik. Approved the final version of the work on behalf of all authors: Lawton. Supervision: Lawton.

## Supplemental Information

### Patient Informed Consent

The necessary patient informed consent was obtained in this study.
